# The Lord Mayor and Hospital Saturday

**Published:** 1913-10-11

**Authors:** David Burnett

**Affiliations:** Lord Mayor. Mansion House, London, E.C.


### EDITOR'S LETTER-BOX.

### The Lord Mayor and Hospital Saturday.

*To the Editor of* THE HOSPITAL.

SIR,—As President, and in accordance with the practice of my predecessors now extending over forty years, I beg to make an urgent appeal on behalf of the Hospital Saturday Fund. The work and objects of the Fund are so well known that I need only remind your readers that it aims at inducing the industrial classes in particular to support regularly and adequately the Metropolitan hospitals and kindred institutions. It provides hospital, sanatorium, convalescent, dental, ambulance, and surgical appliance benefits, most of which are not available under the National Insurance Act. In 1912 the number of such benefits exceeded 65,000. Since the foundation of the Fund no less than £616,000 has been distributed among the Metropolitan hospitals and dispensaries.

This, surely, is a work deserving of encouragement and support. Unfortunately the income of the Fund this year has diminished, the shrinkage amounting, on an average, to £100 per week. I am anxious to assist in a determined effort on the coming "Hospital Saturday" (October 11) to make up this deficiency. I appeal earnestly to all who have not hitherto given in support of the hospitals to send a donation.

I desire also to draw attention to the family collections which are a special feature this year. His Majesty the King, as Patron of the Fund, has graciously expressed his approval of such collections and his good wishes for their success. It is felt that they will help to impress on children the splendid traditions of this country in its relation to the foundation and maintenance of hospitals and the care of the sick. Donations may be addressed to me at the Mansion House, or to the Treasurer of the Fund, the Hon. Harry Lawson, M.P., 54 Gray's Inn Road, W.C. Family collection sheets may be obtained from the Secretary.—I am, Sir, your obedient servant,

DAVID BURNETT, Lord Mayor.

Mansion House, London, E.C.

[Other letters have had to be held over.]

